# Obesity in pregnancy: infant health service utilisation and costs on the NHS

**DOI:** 10.1136/bmjopen-2015-008357

**Published:** 2015-11-26

**Authors:** Kelly L Morgan, Muhammad A Rahman, Rebecca A Hill, Ashrafunnesa Khanom, Ronan A Lyons, Sinead T Brophy

**Affiliations:** 1 Centre for the Development and Evaluation of Complex Interventions for Public Health Improvement (DECIPHer), School of Social Sciences, Cardiff University, Cardiff, UK; 2 The Farr Institute, College of Medicine, Swansea University, Swansea, UK

**Keywords:** HEALTH ECONOMICS, PUBLIC HEALTH, PAEDIATRICS

## Abstract

**Objective:**

To estimate the direct healthcare cost of infants born to overweight or obese mothers to the National Health Service in the UK.

**Design:**

Retrospective prevalence-based study.

**Setting:**

Combined linked anonymised electronic data sets on a cohort of mother–child pairs enrolled on the Growing Up in Wales: Environments for Healthy Living (EHL) study. Infants were categorised according to maternal early-pregnancy body mass index (BMI): healthy weight mother (18.5≤BMI<25 kg/m^2^; n=342), overweight mother (25≤BMI≤29.9 kg/m^2^; n=157) and obese mother (BMI≥30; n=110).

**Participants:**

609 singleton pregnancies with available health service records and an antenatal maternal BMI.

**Primary outcome measure:**

Total health service utilisation and direct healthcare costs for providing these services in the year 2012–2013. Costs are calculated as cost of the infant (no maternal costs considered) and are related to health service usage from birth to age 1 year.

**Results:**

A strong association existed between healthcare usage cost and BMI (p<0.001). Mean total costs were 72% higher among children born to obese mothers (rate ratio (RR) 1.72, 95% CI 1.71 to 1.73) compared with infants born to healthy weight mothers. Higher costings were attributed to a significantly greater number (RR 1.39, 95% CI 1.04 to 1.84) and duration (RR 1.55, 95% CI 1.37 to 1.74) of inpatient visits and a higher number of general practitioner visits (RR 1.10, 95% CI 1.03 to 1.16). Total mean additional resource cost was estimated at £65.13 for infants born to overweight mothers and £1138.11 for infants born to obese mothers, when compared with infants of healthy weight mothers.

**Conclusions:**

Increasingly infants born to mothers with high BMIs consume additional health service resources in the first year of life; this was apparent across inpatient and general practitioner services. Considering both maternal and infant health service use, interventions that cost less than £2310 per person in reducing obesity early pregnancy could be cost-effective.

**Strengths and limitations of this study**

- Study strengths include the availability of clinically recorded early-pregnancy body mass index (BMI) and linked health records providing a comprehensive account of sociodemographics and clinical data.
- Limitations of this study include the use of a BMI value recorded at a single time point, challenges presented when quantifying health service utilisation, for example, inability to account for community services, and the disregard of indirect and intangible health service costs.
- The observational design cannot indicate causation or account for the effects of unmeasured confounding.

## Introduction

With approximately one in five women presenting as obese at antenatal booking,^1^ obesity is one of the greatest challenges to maternity services throughout the UK. Poor maternal health, for example, poor diet^2^ ^3^ and low physical activity levels,^4^ has been shown to predispose infants to adverse health outcomes in later life,^5^ through exposure to an adverse environment in uteri. Moreover, obesity during pregnancy is associated with an increased likelihood of overweight in the offspring,^6^ and in turn overweight children are more likely to become overweight or obese adults.^7^ There is a clear need to target the weight of mothers before pregnancy as this offers not only an avenue for increasing maternal well-being but also a mode of tackling childhood obesity levels.

Considerable research efforts have been made to quantify the amount of capital which can be used to develop effective policies and preventative obesity strategies. Research to date has shown that obesity in adult populations^8^ ^9^ and throughout pregnancy^10^ ^11^ is associated with increasing direct healthcare costs. In agreement, we previously found that obese women place increasing strain on the National Health Service (NHS) in Wales, accessing higher rates of health services throughout pregnancy when compared with healthy weight counterparts.^12^ Calculating costs associated with health service use throughout pregnancy and 2 months after the birth, our findings showed that obese women utilise an £1200 of NHS resources per pregnancy. However, this particular study did not consider subsequent potential healthcare costs associated with infants born to obese mothers.

Studies examining costs associated with infant health service use in the early years of life are scarce. Two studies^13^ ^14^ have examined neonatal healthcare costs associated with smoking during pregnancy, revealing increasing costs among infants exposed to cigarette smoke in uteri. Other studies have examined costs associated with preterm births.^15–17^ One study has reported estimates of infant healthcare costs associated with maternal pre-pregnancy body mass index (BMI), within a 90-day period following birth.^18^ The authors reported that maternal obesity was not associated with excess hospital costs for infants; conversely, infants born to underweight mothers revealed significantly higher costs in comparison to healthy weight pregnancies. This study, however, was limited to inpatient admissions and relied on self-reported BMI. Quantifying the ongoing costs associated with maternal obesity could provide a framework for future interventions and policies aiming to prevent childhood obesity and improve health outcomes for children and mothers.

Using data from a prospective birth cohort study, we aimed to add to the current literature gap, examining follow-up data of infants born to women of varying early-pregnancy BMI. Using a population of mothers from earlier work,^12^ the present study examines infant health service use throughout the first year of life to estimate potential additional costs attributable to maternal obesity during pregnancy in the year 2012–2013 and whether these accrue in the postpartum phase (age 0–7 days), age 1 week to 6 months or from 6 months to 1 year.

## Methods

### Study sample

Data from children born to women who took part in the Growing Up in Wales: Environments for Healthy Living (EHL) birth cohort study^19^ were used for the present study. Pregnant women were recruited from within the Abertawe Bro Morgannwg University (ABMU) NHS Board, in Wales, UK, to participate in the EHL study. Providing health services for a population of 500 000 individuals, ABMU NHS Board is the largest health board in Wales comprising of 18 hospitals and 77 general practices (GPs). Women in the EHL study resided within the City and County of Swansea and surrounding areas; of this population, approximately 95% are Caucasian and 12% of Swansea's local areas are categorised in the 10% most deprived areas of Wales.^20^ Participants (aged 16 and older) were recruited to the study during pregnancy, completed a questionnaire (demographic and socioeconomic data) and provided consent to obtain anthropometric measures. Participants also provided consent for researchers to access medical records (antenatal and postnatal records) for both the mother and the infant.

Exclusion criteria for the present study were multiple births, infants born with a congenital abnormality, those with unobtainable medical records, infants whose mother had a missing early-pregnancy BMI reading, those with an underweight mother during pregnancy and infants who had not reached age 1 by the time of analysis.

### Demographic data

Questionnaire data obtained at baseline provided maternal age at delivery, ethnicity, parity, maternal working status, smoking and alcohol consumption (yes or no) during pregnancy, and psychological distress score (based on Kessler 6 test). Electronic GP medical records were used to identify any diagnoses of a comorbidity within 3 years prior to pregnancy (using the Charlson Index^21^) and to substitute smoking data from missing questionnaire data. The National Community Child Health Database (NCCHD) provided a Lower Super Output Area score and associated multiple deprivation indicator, ranking scores from 1 (most deprived) to 5 (least deprived)) for each mother–child pair and birth data (child gender, birth weight and gestational age).

### BMI categories

Infants were stratified according to maternal early-pregnancy BMI values, which were obtained from antenatal records (calculated by a midwife around 12 weeks gestation). For the purpose of this study, three groups were formed: infants born to healthy weight mothers (18.5≤BMI<25 kg/m^2^), infants born to overweight mothers (25≤BMI≤29.9 kg/m^2^) and infants born to obese mothers (BMI≥30 kg/m^2^).

### Health services use data

Following on from our previous study examining maternal health service use,^12^ we aimed to obtain the same measures of health service use and apply costs concerning the infant. Study methods have previously been described elsewhere when calculating costs for health service use of the mother.^12^ Infant medical records were extracted from birth to age 1 year using the Secure Anonymised Information Linkage (SAIL) databank developed at Swansea University.^22^ We examined GP Read codes enabling us to quantify the number of GP visits, the number of medications prescribed by a GP, the number and length of each inpatient appointment (totalling the number of days for each inpatient admission), and the number of outpatient appointments.

To capture health service use relating to GP visits, Read codes were extracted from the primary care data set. Using a previously adopted method,^12^ we calculated the number of GP visits and also extracted all medications Read codes (indicated by Read codes starting in lower case letters a–z) which had been prescribed throughout the study period (2012–2013). The Patient Episode Database for Wales (PEDW) provided data of any inpatient or outpatient visits across the study period. Data extracted included date of admission spell, date of discharge (in cases of inpatient data) and specialty code for each spell. For each visit, a distinct record was extracted; therefore, it was only possible for an infant to have one record of an outpatient appointment on any given date.

### Economic analysis

In order to compare the mean difference in healthcare costs for each group of infants, specific costs were applied to each health service visit. All healthcare costs are related to the infant only and do not consider any related healthcare use of the mother. For each inpatient and outpatient visit, a specialty unit cost was applied to the patient event. Specialty costings were provided by the Welsh Costing Return (WCR) 2011–2012, and costs are fully inclusive of all medications, treatments and operations an individual might receive during a patient event. Thereafter, the unit cost was multiplied by the number of days the infant spent under that specialty for each inpatient visit. To calculate costs associated with GP visits, it was assumed that each visit involved lasted approximately 17.2 min (including direct care staff costs and qualifications), and a unit cost was provided by the Unit Costs of Health and Social Care 2012.^23^ In order to apply costings to prescriptions, each medication was assigned a unit cost as provided by the British National Formulary November 2011,^24^ relevant to the medication type and dosage. Read codes relating to vaccination were excluded.

### Statistical analyses

Mother–child pairs participating in the Growing Up in Wales birth cohort that were eligible for inclusion in the present study and were divided into three groups, according to maternal early-pregnancy BMI: healthy weight (BMI between 18.5 and 24.9 kg/m^2^), overweight (BMI between 25 and29.9 kg/m^2^) and obese (BMI≥30 kg/m^2^). Demographic characteristics of mother–child pairs were examined and reported according to early-pregnancy BMI group. To identify significant differences in baseline characteristics between all three groups, Kruskal-Wallis tests for categorical measures and one-way analysis of variance for continuous measures were carried out. Student t tests were conducted to detect any difference in characteristics between those mother–child pairs included in the study and mother–child pairs excluded due to lack of maternal BMI data. The mean and SD for each hospital service use and cost are reported for the healthy weight group. Poisson regression models were carried out to report incident rate ratios (RRs) and 95% CIs for health service use and associated costs, comparing findings of overweight and obese groups relative to healthy weight. As the cost data were skewed, further parametric modelling was not carried out. STATA V.12.1 was used for all statistical analyses, and statistical significance was set at p<0.05 throughout.

## Results

### Demographics

Among the total study population, 609 (83.7%) mother–child pairs met the study inclusion criteria. The average age of mothers at birth was 29.3 years and infants had a mean birth weight and gestational age of 3.4 kg and 39.5 weeks, respectively. In terms of deprivation scores, one-fifth of the total study sample were from the most deprived category (n=121) and one-quarter (n=152) from the least deprived category.

**Figure 1:**
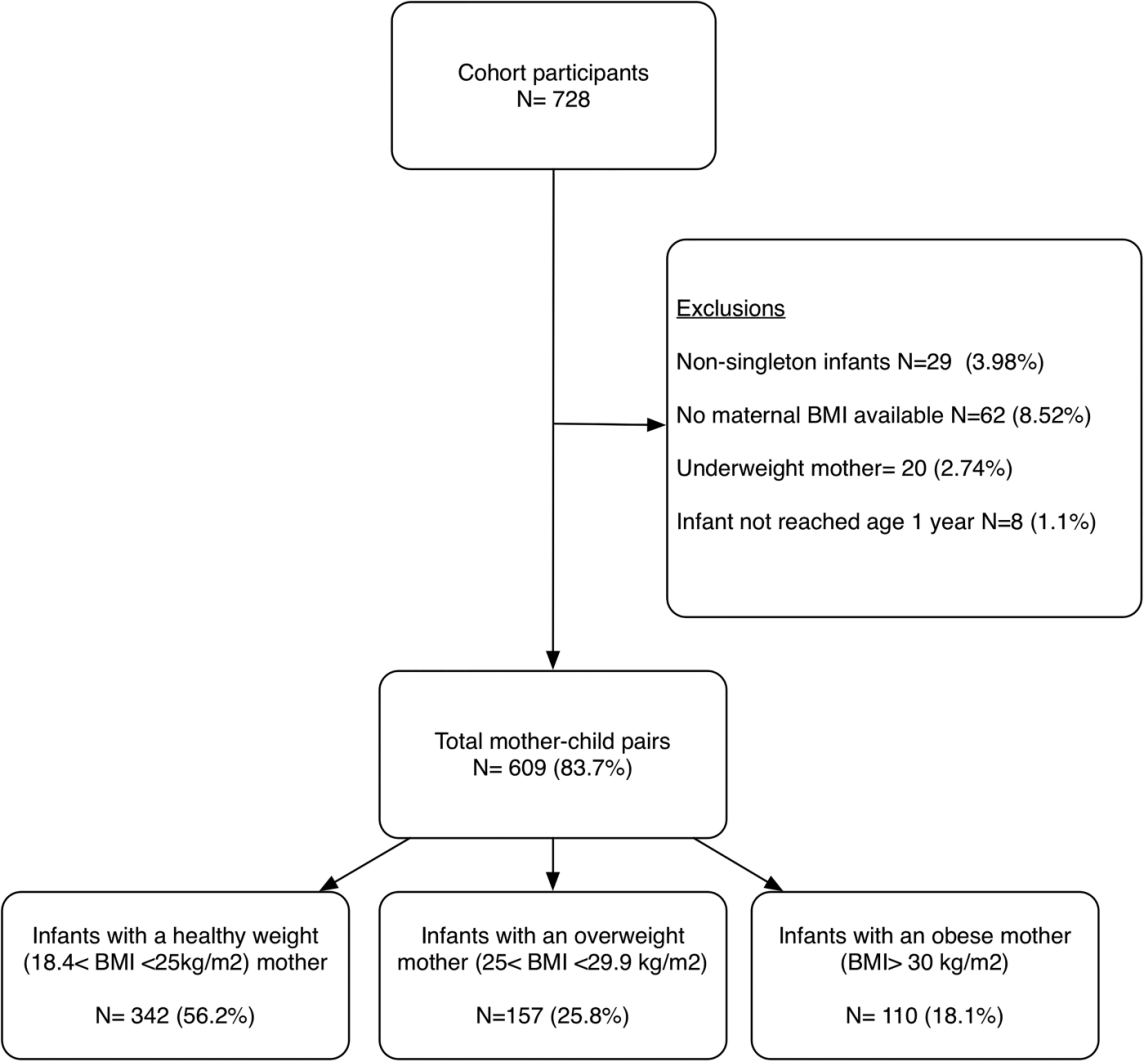
A flow diagram depicting participant involvement within the study (BMI, body mass index).

Figure 1 highlights the process for obtaining the study population, and descriptive statistics for all three groups of mother–child pairs are shown in table 1. Over half of mothers had a healthy weight (BMI between 18.5 and 24.9 kg/m^2^) during early pregnancy and approximately 44% of mothers were overweight or obese (BMI>25 kg/m^2^). Mothers who were obese were more likely to be affluent (signified by Lower Super Output Area (LSOA) deprivation score), have at least one previous child and deliver a macrosomic infant. No significant differences were shown between the number of women smoking and reporting alcohol consumption during pregnancy between the three groups. Mothers excluded because of missing early-pregnancy BMI data were of similar age (29.3 vs 28.6, p=0.42) and deprivation (3.0 vs 3.1, p=0.38) to those included within the study. Their infants also displayed similar birth weights (3.38 vs 3.43, p=0.45) and gestational ages (39.48 vs 39.53, p=0.83) as those infants included.

**Table 1:** Characteristics of the total study population of mother–child pairs (N=609)

Characteristics (n)	BMI (kg/m^2^)			p Value
	18.5–24.9	25–29.9	≥30	
Number of participants	342	157	110	
*Maternal*	n (%)	n (%)	n (%)	
Age at delivery (years) (n=602) (range 17–44)				
<20	14 (4.1)	6 (3.9)	5 (4.7)	NS
20–30	154 (45.4)	76 (48.7)	52 (48.6)	
30–40	159 (46.9)	70 (44.9)	45 (42.1)	
≥40	12 (3.5)	4 (2.6)	5 (4.7)	
Ethnic group (n=329)				
White/European	191 (91.4)	69 (87.3)	39 (95.1)	NS
Other	18 (8.6)	10 (12.7)	2 (14.3)	
Working status (n=301)				
Employed	92 (82.1)	56 (69.2)	74 (43.5)	***
Unemployed	9 (8)	12 (14.8)	11 (10.2)	
Homemaker	4 (3.6)	7 (8.6)	19 (17.6)	
Other	7 (6.3)	6 (7.4)	4 (3.7)	
Deprivation score (588)				
1(most deprived)	82 (24.8)	27 (17.7)	11 (10.6)	***
2	69 (20.9)	31 (20.2)	14 (13.5)	
3	49 (14.8)	20 (13.1)	16 (15.4)	
4	57 (17.2)	38 (24.8)	22 (21.2)	
5 (least deprived)	74 (22.3)	37 (24.2)	41 (39.4)	
Parity (n=353) (range 0–4)				
0	108 (49.1)	33 (37.9)	15 (32.6)	**
1+	112 (50.9)	54 (62.1)	31 (67.4)	
Co morbidity within 3 years prior‡ (n=609)	19 (5.5)	15 (9.4)	10 (9.3)	*
Smoker (n=554)	82 (26.7)	36 (24.7)	33 (32.7)	NS
Missing data	35 (10.2)	11 (7.0)	9 (8.2)	
Alcohol consumption (n=333)	131 (39.4)	39 (37.4)	39 (48.2)	NS
Missing data	131 (38.3)	76 (48.4)	69 (62.7)	
Distress score† ≥12 (n=329)	5 (4.6)	4 (5.1)	5 (4.7)	*
Missing data	134 (39.2)	77 (49.0)	69 (62.7)	
*Infant*				
Male (609)	190 (55.6)	71 (45.2)	56 (59.1)	*
Gestational age (597) (range 30–43), weeks				
<37	10 (2.9)	10 (6.5)	2 (1.9)	***
37–41	312 (92.9)	133 (86.4)	99 (92.5)	
≥42	14 (4.2)	11 (7.1)	6 (5.6)	
Birth weight (602) (range 2.2–5.1), kg				
<2.5	10 (2.9)	4 (2.6)	4 (3.7)	**
2.5–4	288 (85.0)	139 (89.1)	85 (79.4)	
>4	41 (12.1)	13 (8.3)	18 (16.8)	

***p<0.001, **p<0.05, *p<0.1.

†Based on score of total Kessler 6 test.

‡Based on the Charlson index.

BMI, body mass index; NS, not significant.

### Health service utilisation

The usage rate for all healthcare services, with the exception of outpatient visits, was significantly higher for infants born to obese mothers when compared with infants born to healthy weight mothers (table 2). Specifically, infants born to obese mothers experienced a 39% higher rate of inpatient visits and a 55% increase in the duration of inpatient visits. Examining GP data, a 10% higher visit rate and 10% higher prescription of medications were shown among infants born to obese mothers compared with healthy weight mothers. No significant differences were observed when comparing the overweight group to healthy weight mothers.

**Table 2:** Infant health service use in relation to maternal early-pregnancy BMI (kg/m^2^) by type of service

Health service	BMI 18.5–24.9	BMI 25–29.9		BMI≥30	
	Mean (SD)	Mean (SD)	Rate ratio* (95% CI)	Mean (SD)	Rate ratio* (95% CI)
Inpatient	1.7 (2.1)	2.1 (1.7)	1.23 (0.94 to 1.61)	2.4 (1.9)	1.39 (1.04 to 1.84)
Inpatient duration	1.1 (2.2)	1.0 (1.7)	1.11 (0.89 to 1.38)	1.7 (3.9)	1.55 (1.37 to 1.74)
Outpatient	1.6 (3.7)	1.5 (2.9)	0.93 (0.79 to 1.08)	1.5 (3.3)	0.96 (0.81 to 1.14)
GP	14.1 (7.8)	14.0 (6.3)	0.99 (0.95 to 1.04)	15.5 (9.7)	1.10 (1.03 to 1.16)
Medication	6.5 (9.2)	5.9 (8.7)	0.99 (0.95 to 1.05)	7.2 (8.6)	1.10 (1.04 to 1.16)

*Rate ratios based on mean utilisation of health services over 12 months relative to participants with a maternal pre-pregnancy BMI of 18.5–24.9 kg/m^2^.

BMI, body mass index; GP, general practice.

### Cost evaluation

A strong association between mean total costs and maternal BMI was evident, with infants born to obese mothers utilising on average 72% higher resource costs (p<0.01). Table 3 provides a breakdown of mean resource costs by health service. As shown, infants born to obese mothers had higher resource costs for each health service with the exception of outpatient visits and prescribed medications. Infants born to overweight mothers also had higher mean total costs; however, significantly lower costs were observed for outpatient visits, GP visits and prescribed medications.

**Table 3:** Cost of infant health service use in relation to maternal early-pregnancy BMI by type of service (£/12 months)

Health service	BMI 18.5–24.9	BMI 25–29.9		BMI≥30	
	Mean (SD)	Mean (SD)	Rate ratio* (95% CI)	Mean (SD)	Rate ratio* (95% CI)
Inpatient	641.3 (2525.6)	739.5 (1815.9)	1.15 (1.15 to 1.16)	1711.5 (6423.1)	2.67 (2.65 to 2.69)
Outpatient	177.08 (517.7)	145.0 (337.6)	0.85 (0.83 to 0.86)	173.5 (427.4)	0.98 (0.96 to 1.0)
GP	799.2 (372.5)	788.1 (312.71	0.97 (0.97 to 0.98)	902.3 (48.4)	1.13 (1.12 to 1.14)
Medication	17.9 (18.0)	15.0 (13.6)	0.88 (0.82 to 0.94)	19.0 (17.6)	1.01 (0.93 to 1.09)
Total costs	1572.71 (3147.0)	1639.0 (2118.4)	1.04 (1.03 to 1.05)	2712.2 (6547.0	1.72 (1.71 to 1.73)

*Rate ratios based on mean health service costs over 12 months relative to participants with a maternal early-pregnancy BMI of 18.5–24.9 kg/m^2^.

BMI, body mass index; GP, general practice.

Table 4 provides a breakdown of resource costs for the three periods: postpartum, age 1 week to 6 months and age 6–12 months. As shown, infants born to obese mothers accrued higher mean costs for all three periods, with greatest costs demonstrated in the postpartum phase (274% higher than infants born to healthy weight mothers). Conversely, infants born to overweight mothers accrued significantly lower costs in the first and last period while revealing higher costs in early infancy. Examining inpatient appointments, infants born to obese mothers accessed a significantly higher number of surgical specialties in comparison to the other two groups (9% vs 0.5%), all of which occurred after the postpartum stage (later than age 1 week). No significant differences were evident when looking at the type of specialty (eg, medical vs surgical vs other) infants accessed for outpatient appointments. Looking specifically at the health services accessed in the first week of life, higher costs in the obese group were attributed to longer inpatient stays (mean duration of healthy weight group, 2.76 (SD 4.2), obese group RR1.74 (95% CI 1.41 to 2.14)).

**Table 4:** Cost of infant health service in relation to maternal early-pregnancy BMI by time period (£/12 months)*

Time frame	BMI 18.5–24.9	BMI 25–29.9		BMI≥30	
	Mean (SD)	Mean (SD)	Rate ratio* (95% CI)	Mean (SD)	Rate ratio* (95% CI)
0–7 days	242.84 (1236.7)	147.7 (594.9)	0.61 (0.60 to 0.62)	664.1 (2958.9)	2.74 (2.7 to 2.76)
1 week to 6 months	927.0 (1609.8)	1159.1 (1737.4)	1.25 (1.24 to 1.26)	1492.6 (3040.5)	1.61 (1.60 to 1.62)
6–12 months	405.66 (809.2)	333.2 (389.9)	0.82 (0.81 to 0.83)	555.3 (1378.2)	1.37 (1.36 to 1.38)

*Based on inpatient, outpatient and general practice costs (including prescriptions).

BMI, body mass index.

Examining GP visits, infants born to obese mothers had a significantly lower cost in the postpartum phase (mean cost of healthy weight group, 41.5 (SD 42.2), obese group RR 0.96 (95% CI 0.93 to 0.99)) when compared with the healthy weight group. Significantly higher costings were observed during the periods 1 week to 6 months (healthy weight group, 487.4 (SD 247.6), obese group RR 1.11 (95% CI 1.10 to 1.12)) and 6–12 months (healthy weight group, 216.5 (SD 205.1), obese group RR 1.09 (95% CI 1.08 to 1.11)). In comparison to the healthy weight group, higher costings were also apparent for infants born to overweight mothers in the 1-week to 6-month period (RR 1.02 (95% CI 1.01 to 1.03)) but lower within the 6–12-month period (RR 0.93 (95% CI 0.92 to 0.94)). Over the 1-year period, the mean total cost difference was estimated at £65.13 for infants born to overweight mothers and £1138.11 for infants born to obese mothers when compared with infants of healthy weight mothers.

## Discussion

In an earlier study, we reported increased health service use among mothers with early-pregnancy BMI values at or above 30 kg/m^2^, with economic analyses revealing that obese women utilise additional NHS resources, amounting to on average £1172 more per pregnancy than healthy weight women.^12^ These findings highlighted additional expenditure which might be better spent on interventions which enable women to enter pregnancy with a healthy BMI. The present study sought to estimate whether additional costs are also accrued as a result of health services accessed by infants born to obese women.

Our findings demonstrate that infants exposed to an obesogenic environment during uteri use on average an extra £1138 in NHS resources throughout the first year of life in comparison to infants born to mothers with a healthy BMI. Looking specifically at the type of health service accessed, all services, with the exception of outpatient appointments, were higher for infants of obese mothers. Breaking costs down into time periods, the greatest difference in infant costs occurred within the postpartum phase. One possible explanation for these findings is that infants born to obese mothers have longer inpatient stays during the postpartum phase as a result of delivery complications and increasing birth weights.^25^ As with all observational studies, however, it is impossible to rule out the potential for unmeasured confounding. The availability of clinically recorded BMI values is one of the evident strength of the present study. Using a cohort of mother–child pairs who had participated in data collection throughout pregnancy enabled clinically recorded BMI values to be extracted from antenatal records, reducing problems associated with misclassification from recall bias.^26^ The availability of linked health data made it possible to document infant's health service use and include a comprehensive account of sociodemographics and clinical data, for example, smoking status during pregnancy, deprivation scores and comorbidity data. Within our analyses, we did not adjust for social deprivation as our earlier analyses showed no significant associations.^12^ There are, however, other limitations which must also be acknowledged. First, we lack full ethnicity data due to incomplete or missing participant questionnaires. As ethnicity is often not captured with anonymised linked health data or coded with a default value, we were unable to gather these data retrospectively. Second, as highlighted in our earlier study,^12^ we are unable to account for costs associated with community services such as health visitor contacts. It is likely that our cost estimates are conservative if the same pattern was replicated in community services. Using anonymised health data also limits our certainty surrounding the type of contact infants had with a service, for example, appointment with a general practitioner versus a general nurse. This limitation has been discussed in greater detail previously.^12^ A further consideration when interpreting the study findings is that maternal BMI was assessed at the time of booking for antenatal care. Our findings therefore do not reflect gestational weight gain or loss and the possibilities for women to alter BMI categories during pregnancy. Lastly, our findings are restricted to a small sample of women residing within an area of South Wales who opted to take part within our study. The generalisability of our findings to wider populations is therefore uncertain, especially due to the predominantly white population, and a large proportion of affluent women residing within the obese group. Such factors could influence mother's help-seeking behaviours for infants.^27^

The findings from this study are coherent with and expand on previous work that reports increasing health service use and costs among offspring exposed to adverse environments in uteri. Multiple studies have reported increasing health service use and associated costs among infants exposed to cigarette smoke in uteri.^13^ ^28^ One study estimated that infants born to mothers who smoked 20 or more cigarettes per day, cost £462 extra through increased inpatient appointments when compared with infants not exposed to smoke during pregnancy.^13^ Previous studies examining perinatal outcomes in pregnancies complicated by obesity have reported approximately 3.5 times higher neonatal admissions in comparison to women of healthy weights.^29–31^ Observing significant findings among infants born to massively obese mothers (>300 pounds), Perlow *et al*^30^ noted that once women with diabetes and/or hypertension were excluded from analyses, no differences remained in the number of neonatal admissions. Limited studies, however, have published estimates on the magnitude of costs associated with increased child health service use according to maternal BMI. A recent Australian-based study^18^ analysed birth and neonatal intensive care admissions over a 90-day period following birth and reported infant costs according to maternal early-pregnancy BMI groupings. On the contrary to our findings, the authors observed a significantly shorter length of hospital stay among infants born to overweight and obese women and did not find any excessive offspring costs. In addition to differing population characteristics and economic approaches, the disparity in findings could be a result of the different time periods observed, for example, the present study examined costs over a 1 year period, whereas Watson *et al*^18^ examined a 90-day period. Furthermore, the authors highlight that their findings are reliant on self-reported maternal BMI values; therefore, they cannot be certain that BMI values have not been underestimated or overestimated.

Integrating our earlier findings^12^ with the present results, we estimate that obesity during pregnancy costs on average £2310 extra from the time of conception to the infants first birthday. With 1 in 5 women attending prenatal care being classed as obese in UK^32^ and 778 805 births in the UK during 2013,^33^ ^34^ this equates to an additional resource use of £359 807 910 on healthcare services. Future work needs to examine the casual pathways to explain why we are observing increased usage and costs.

This is one of, if not the first, UK-based study to assess infant health service use and associated costs according to maternal early-pregnancy BMI. Our findings showed a significant increase in health service use and associated costs for infants born to obese mothers. In conjunction with our previous study,^12^ the present study adds an economic dimension to the importance of promoting healthy weights among women of reproductive ages. The findings from this study should help inform policy makers and stimulate the design of cost-effective interventions to prevent maternal obesity.
